# The Association between Suicidal Ideation and Subtypes of Comorbid Insomnia Disorder in Apneic Individuals

**DOI:** 10.3390/jcm13195907

**Published:** 2024-10-03

**Authors:** Matthieu Hein, Benjamin Wacquier, Matteo Conenna, Jean-Pol Lanquart, Camille Point

**Affiliations:** 1 Service de Psychiatrie et Laboratoire du Sommeil, Hôpital Universitaire de Bruxelles, Université libre de Bruxelles, ULB, 1070 Bruxelles, Belgium; benjamin.wacquier@hubruxelles.be (B.W.); matteo.conenna@hubruxelles.be (M.C.); secmed.psy.erasme@hubruxelles.be (J.-P.L.); camille.point@hubruxelles.be (C.P.); 2 Laboratoire de Psychologie Médicale et Addictologie (ULB312), Université Libre de Bruxelles, ULB, 1020 Bruxelles, Belgium

**Keywords:** suicidal ideation, insomnia disorder, obstructive sleep apnea syndrome, polysomnography

## Abstract

**Background/Objectives**: Given the existence of higher suicidality in apneic individuals, this study aimed to determine the potential role played by subtypes of the comorbid insomnia disorder (CID) in the occurrence of suicidal ideation for this specific subpopulation. **Methods:** To perform our analyses, 1488 apneic individuals were retrospectively extracted from the Sleep Laboratory hospitalization register. Only apneic individuals with suicidal ideation highlighted during the psychiatric interview and/or with a score ≥1 on item G of the Beck Depression Inventory confirmed during the clinical interview were included in the group with suicidal ideation. The likelihood of suicidal ideation associated with CID subtypes was investigated using logistic regression analyses. **Results:** The prevalence of suicidal ideation was 9.3% in our sample of apneic individuals. After hierarchically introducing the significant confounders for adjustment, multivariate logistic regression analyses demonstrated that unlike short sleep duration alone and CID without short sleep duration, the likelihood of suicidal ideation was only higher for CID with short sleep duration in apneic individuals. **Conclusions:** Thus, we highlighted in this study that CID with short sleep duration could play a major role in higher suicidality for apneic individuals, which seems to require systematic screening and appropriate treatment of this comorbid sleep disorder to enable better management of suicidal risk in this specific subpopulation.

## 1. Introduction

Suicide is a significant challenge to public health policies in many countries. Indeed, around 800,000 suicides occur annually across the world and suicide mortality is one of the leading causes of death in young adults with an age <30 years [1]. Moreover, in the majority of these individuals committing suicide, the occurrence of suicidal acting is generally preceded by different steps, the first of which is the development of suicidal ideation (SI) [2]. However, the annual and lifetime prevalence of these SIs is, respectively, 2.0% and 9.2% in the general population, which indicates that the occurrence of this first step of the suicidal plan is not a problem to be neglected [3,4]. In addition, some specific features of these SIs (degree of chronicity and planning) may have a major impact on the evolution of the suicidal plan with higher risk of suicidal acting [5,6]. However, given the data available in the literature, one of the subpopulations at high risk of SI could be apneic individuals since the prevalence of SI is higher in this particular subpopulation (9.7–20.1%) than the general population [7,8]. In addition, it has been demonstrated that the SI severity was correlated with the severity of obstructive sleep apnea syndrome (OSAS) [9]. Thus, following these different elements, the realization of additional studies is essential to better identify the potential cofactors involved in this particular relationship between SI and OSAS in order to enable a better understanding of higher suicidality in this specific subgroup of patients.

Based on available studies, insomnia disorder appears to be associated with higher risk of SI both for the general population and for some subpopulations [10,11]. Furthermore, the prevalence of SI seems to be higher (25–32%) among insomniac individuals than in the general population [12]. Additionally, the SI severity has been shown to increase with the severity of insomnia symptoms [13]. However, although insomnia disorder is a frequent comorbidity in apneic individuals [14,15], a limited number of studies have investigated the role played by this comorbid sleep disorder in the occurrence of SI for this specific subgroup of patients [16]. In addition, in these few available studies [16], insomnia complaints and SI were only assessed in apneic individuals by self-questionnaires without confirmation during clinical interviews with healthcare professionals, which may significantly limit the interpretation of their results. Furthermore, despite evidence supporting a different impact of insomnia subtypes (categorized according to sleep duration) on suicidality [17], no study has investigated the potential role played by short sleep duration (SSD) associated with insomnia disorder in the risk of SI in apneic individuals. Indeed, despite a non-negligible prevalence of SSD associated with insomnia disorder in apneic individuals [14], available studies have only focused on other subpopulations [17], which may prevent the generalization of their results to the specific subpopulation of apneic individuals. In this context, after taking into account the limitations of these previous studies (absence of clinical interviews for diagnosis of insomnia disorder and assessment of SI), there seems to be a major interest in investigating the likelihood of SI associated with insomnia subtypes based on sleep duration in apneic individuals to allow better management of suicidal risk for this specific subgroup of patients.

This study aimed to demonstrate that after adjustment for classic risk factors associated with suicidality, there is a different impact of insomnia subtypes categorized according to sleep duration on the likelihood of SI in apneic individuals. In addition, alongside this main objective, this study had the additional objective of investigating the prevalence of SI in apneic individuals. Our hypothesis was that unlike comorbid insomnia disorder (CID) without SSD, only CID with SSD promotes a more frequent occurrence of SI for apneic individuals. Furthermore, in order to allow the best possible scientific rigor in the testing of this hypothesis, the limitations of other previous studies available in the literature were taken into account thanks to the presence of clinical interviews for the diagnosis of CID and the assessment of SI in apneic individuals included for this study. The goal of this study was therefore to provide reliable data to health professionals on the likelihood of SI associated with insomnia subtypes based on sleep duration in apneic individuals to enable the development of new suicide risk prevention strategies for this specific subgroup of patients.

## 2. Materials and Methods

### 2.1. Selection of Apneic Individuals

From the register of hospitalizations for polysomnography at the Sleep Laboratory of the Brussels University Hospital between 1 January 2002 and 31 December 2020, 1488 apneic individuals were retrospectively selected for this study. The inclusion criteria applied were the presence of OSAS according to the diagnostic criteria of the American Academy of Sleep Medicine (obstructive apnea–hypopnea index [OAHI] ≥ 5/h) [18], absence of severe psychiatric disorders (including active suicidal ideations with high risk of suicidal acting), absence of substance use disorders, and absence of pregnancy, whereas the exclusion criteria applied were the presence of acute and/or uncontrolled medical pathologies, presence of some sleep disorders (parasomnia, narcolepsy, primary hypersomnia, predominantly central sleep apnea syndrome), presence of OSAS treatment already initiated, presence of malformations of the thoracic cage or the orofacial sphere, and presence of acquired or congenital brain lesions. For this study, given the existence of data already validated in the literature in favor of higher suicidality associated with OSAS [7,19,20,21,22,23,24,25], we decided to focus our recruitment only on apneic individuals since our aim was to investigate the potential involvement of insomnia subtypes categorized according to sleep duration in higher suicidality for this specific subgroup of patients. Indeed, after being based on other studies using this similar approach [8,16], this choice of recruitment focused solely on apneic individuals for the design of our study aimed to determine whether some insomnia subtypes categorized according to sleep duration were specific risk factors for higher suicidality within this particular subpopulation. Finally, the description of the outpatient care pathway for these apneic individuals before their admission to the Sleep Laboratory of the Brussels University Hospital is available in the Supplementary Data—Annex S1.

### 2.2. Method

**Figure 1:**
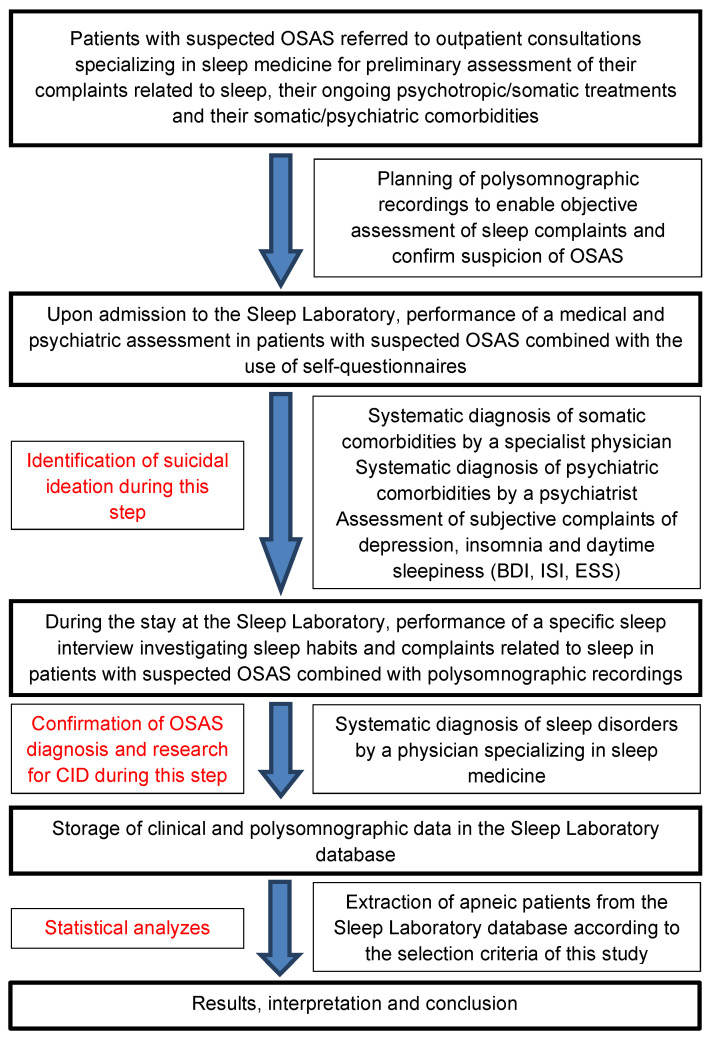
Organizational diagram of study. OSAS = obstructive sleep apnea syndrome, ESS = Epworth Sleepiness Scale, BDI = Beck Depression Inventory, ISI = Insomnia Severity Index, CID = comorbid insomnia disorder.

The organizational diagram of this study is available in Figure 1.

#### 2.2.1. Medical and Psychiatric Assessment of Apneic Individuals

During their stay in the specialized unit for polysomnographic recordings, all these apneic individuals underwent a complete clinical evaluation subdivided into three parts:

(1) A systematic diagnosis of their potential somatic comorbidities by a physician assigned to the Sleep Laboratory thanks to a medical check-up including a review of the medical record, a structured medical interview, a physical examination, and additional tests (urine analyses, blood test, electrocardiogram, and daytime electroencephalogram).
(2) A comprehensive diagnosis of their possible psychiatric comorbidities (including assessment of suicidality [thoughts of death, passive or active SI, history of suicide attempts, non-suicidal self-injury, and suicide equivalents]) by a physician specialized in psychiatry attached to the Sleep Laboratory using a standardized psychiatric interview structured according to diagnostic criteria of the DSM-IV-TR (before 2013) and DSM 5 (after 2013) [26,27].
(3) A systematic assessment of the severity of their self-reported symptoms of insomnia, depression, and daytime sleepiness using self-questionnaires (Insomnia Severity Index, 13-item Beck Depression Inventory, and Epworth Sleepiness Scale) described in the Supplementary Data—Annex S2 [28,29,30].

Based on this complete clinical assessment, SIs were considered to be present in apneic individuals recruited for this study when they had been identified during the semi-structured psychiatric interview and/or when the score on item G of the Beck Depression Inventory (reduced to 13 items) was ≥1 with confirmation by the clinical interview [31,32,33].

#### 2.2.2. Sleep Assessment of Apneic Individuals

After this clinical evaluation, all these apneic individuals benefited from a complete sleep assessment subdivided into two parts:

(1) A systematic screening of their potential symptoms suggestive of the main sleep disorders by a physician specialized in sleep medicine thanks to a specific sleep interview investigating sleep habits and complaints related to sleep.
(2) A polysomnographic recording with a montage meeting international recommendations (Supplementary Data—Annex S3) that was carried out under the usual stay conditions of the Sleep Laboratory of the Brussels University Hospital (Supplementary Data—Annex S4) and was scored visually by specialized technicians according to the scoring criteria of the American Academy of Sleep Medicine under the supervision of certified somnologists (Supplementary Data—Annex S5) [34,35,36,37].

Based on this complete sleep assessment, a confirmation of the OSAS diagnosis, a determination of OSAS severity (mild [OAHI 5–14/h], moderate [OAHI 15–29/h], and severe [OAHI ≥ 30/h]), and a systematic screening for potential comorbid sleep disorders according to the diagnostic criteria validated in the literature (insomnia disorder, SSD, restless legs syndrome, and moderate to severe periodic limb movement disorder [periodic limb movement index ≥ 15/h]) were performed by a physician specialized in sleep medicine in all apneic individuals recruited for this study [38,39,40,41,42].

Finally, a physician specialized in sleep medicine has carried out a classification of insomnia subtypes based on sleep duration in all apneic individuals selected for this study using the following criteria [41,42]:

(1) SSD alone was defined as sleep duration <6 h in the absence of CID;
(2) CID without SSD was defined as the presence of an insomnia disorder according to the diagnostic criteria of the American Academy of Sleep Medicine Work Group (Table 1) comorbid with OSAS characterized by sleep duration ≥6 h;
(3) CID with SSD was defined as the presence of an insomnia disorder according to the diagnostic criteria of the American Academy of Sleep Medicine Work Group (Table 1) comorbid with OSAS characterized by sleep duration <6 h.

**Table 1:** Research Diagnostic Criteria for insomnia disorder [41].

Criteria	
A	The individual reports one or more of the following sleep-related complaints:
	1. difficulty initiating sleep
	2. difficulty maintaining sleep
	3. waking up too early
	4. sleep that is chronically nonrestorative or poor in quality
B	The above sleep difficulty occurs despite adequate opportunity and circumstances for sleep
C	At least one of the following forms of daytime impairment related to the nighttime sleep difficulty is reported by the individual:
	1. fatigue/malaise
	2. attention, concentration, or memory impairment
	3. social/vocational dysfunction or poor school performance
	4. mood disturbance/irritability
	5. daytime sleepiness
	6. motivation/energy/initiative reduction
	7. proneness for errors/accidents at work or while driving
	8. tension headaches and/or gastrointestinal symptoms in response to sleep loss
	9. concerns or worries about sleep
Minimum duration of insomnia complaints	At least one month

### 2.3. Statistical Analyses

Statistical analyses were carried out using version 14 of the Stata software. In order to perform our analyses, the sample of apneic individuals of this study was subdivided into two groups: a group without suicidality and a group with suicidality. Only apneic individuals with SI highlighted during the semi-structured psychiatric interview and/or with a score ≥1 on item G of the Beck Depression Inventory (reduced to 13 items) confirmed during the clinical interview were included in the group with suicidality [31,32,33].

After checking the data distribution and the equality of variances, the application of parametric tests was not possible for most of the continuous data in this study, which required the use of non-parametric tests. In this context, for continuous data, descriptive analyses were carried out using medians with their P25-P75 and comparison analyses were performed using Wilcoxon tests. Furthermore, categorical data were described by percentages and compared between groups of apneic individuals by Chi^2^ tests.

Univariate logistic regression analyses were carried out to investigate the likelihood of SI associated with CID (categorized: no, SSD alone, CID without SSD, CID with SSD) and potential confounders (Supplementary Data—Annex S6). Afterwards, multivariate logistic regression analyses were used to adjust the likelihood of SI associated with CID through a hierarchical introduction of significant confounders identified during the univariate analyses. The specificity and adequacy of the final multivariate logistic regression model were checked, respectively, by the Link test and by the Hosmer and Lemeshow test.

The statistical significance cut-off was set at a *p*-value < 0.05 for this study.

## 3. Results

### 3.1. Polysomnographic Data (Table 2)

Sleep latency (42.0 [20.0–80.0] vs. 24.5 [13.0–48.5], *p*-value < 0.001), percentage of stage 3 (5.2 [0.3–14.5] vs. 2.8 [0.2–8.6], *p*-value = 0.011), and REM latency (98.5 [64.0–174.0] vs. 81.5 [59.0–123.3], *p*-value < 0.001) were higher in apneic individuals with SI than in those without SI. Furthermore, apneic individuals with SI had a shorter sleep period time than those without SI (433.5 [404.3–479.5] vs. 453.5 [413.3–485.5], *p*-value = 0.033). Finally, the other polysomnographic variables did not present significant differences between the two groups of apneic individuals.

**Table 2:** Polysomnographic data (*n* = 1488).

	Whole Sample (*n* = 1488)	Subjects without SI (*n* = 1349)	Subjects with SI (*n* = 139)	*p*-Value
Sleep latency (min)	25.2 (13.0–51.5)	24.5 (13.0–48.5)	42.0 (20.0–80.0)	<0.001
Sleep efficiency (%)	77.9 (67.8–85.1)	78.2 (68.0–85.3)	75.8 (65.7–83.8)	0.062
Sleep period time (min)	452.3 (412.0–485.5)	453.5 (413.3–485.5)	433.5 (404.3–479.5)	0.033
Total sleep time (min)	381.0 (334.0–426.3)	381.5 (334.0–427.5)	374.5 (331.5–415.0)	0.399
% stage 1	8.7 (6.0–12.4)	8.7 (6.0–12.4)	8.6 (5.4–12.0)	0.465
% stage 2	53.8 (47.0–59.8)	53.8 (47.2–59.7)	53.5 (45.6–61.2)	0.854
% stage 3	2.9 (0.2–9.2)	2.8 (0.2–8.6)	5.2 (0.3–14.5)	0.011
% REM sleep	15.7 (11.4–20.1)	15.8 (11.6–20.2)	14.5 (8.9–20.0)	0.056
REM latency (min)	83.0 (59.5–126.7)	81.5 (59.0–123.3)	98.5 (64.0–174.0)	<0.001
% WASO	13.2 (7.6–21.4)	13.3 (7.7–21.4)	12.5 (5.7–20.3)	0.114
Number of awakenings	32 (22–48)	33 (22–48)	30 (20–47)	0.287
Micro-arousal index	14 (8–23)	14 (8–23)	12 (8–19)	0.067
Apnea–hypopnea index	14 (8–29)	14 (8–30)	13 (7–23)	0.259
Oxygen desaturation index	6 (2–15)	6 (2–16)	7 (3–15)	0.300
Total time under 90% of SaO_2_ (min)	11.5 (1.0–60.5)	11.7 (1.0–61.7)	9.0 (1.0–48.0)	0.439
PLMS index	1 (0–10)	2 (0–10)	1 (0–10)	0.644
	Median (P25–P75)	Median (P25–P75)	Median (P25–P75)	Wilcoxon test

SI = suicidal ideation, REM = rapid eye movement sleep, WASO = wake after sleep onset, SaO_2_ = oxygen saturation, PLMS = periodic limb movements during sleep.

### 3.2. Univariate Analyses (Table 3)

The prevalence of SI was 9.3% in our sample of apneic individuals. Gender (*p*-value < 0.001), age (*p*-value = 0.007), use of antidepressants (*p*-value < 0.001), taking benzodiazepine receptor agonists (*p*-value < 0.001), use of other psychotropic drugs (*p*-value = 0.001), cardiometabolic comorbidities (*p*-value = 0.035), excessive daytime sleepiness (*p*-value = 0.015), Beck Depression Inventory scoring without item G > 10 (*p*-value < 0.001), and CID (*p*-value < 0.001) were significantly associated with the occurrence of SI in apneic individuals. Furthermore, apneic individuals with SI had higher scores on the Insomnia Severity Index (16 [13,14,15,16,17,18,19,20] vs. 12 [8,9,10,11,12,13,14,15,16,17], *p*-value < 0.001), the Epworth Sleepiness Scale (11 [7,8,9,10,11,12,13,14] vs. 9 [6,7,8,9,10,11,12,13], *p*-value = 0.001), the 13-item Beck Depression Inventory (13 [9,10,11,12,13,14,15,16,17,18] vs. 3 [1,2,3,4,5,6], *p*-value < 0.001), and the 13-item Beck Depression Inventory without item G (12 [8,9,10,11,12,13,14,15,16,17] vs. 3 [1,2,3,4,5,6], *p*-value < 0.001) than those without SI. Furthermore, other demographic variables did not show significant differences between the two groups of apneic individuals. Finally, the prevalence of SSD alone, CID without SSD, and CID with SSD was, respectively, 20.7%, 24.3%, and 16.6% in our sample of apneic individuals.

**Table 3:** Univariate analyses (*n* = 1488).

Variables	Categories	%	Subjects without SI	Subjects with SI	*p*-Value Chi^2^	OR (CI 95%)	*p*-Value
Gender	Female (*n* = 343)	23.0%	21.5%	38.1%	<0.001	1	<0.001
	Male (*n* = 1145)	77.0%	78.5%	61.9%		0.44 (0.31 to 0.64)	
Age (years)	<65 (*n* = 1314)	88.3%	87.6%	95.7%	0.004	1	0.007
	≥65 (*n* = 174)	11.7%	12.4%	4.3%		0.32 (0.14 to 0.73)	
BMI (kg/m^2^)	<25 (*n* = 289)	19.4%	18.9%	24.5%	0.177	1	0.180
	≥25 and <30 (*n* = 559)	37.6%	38.2%	31.7%		0.64 (0.40 to 1.03)	
	≥30 (*n* = 640)	43.0%	42.9%	43.8%		0.79 (0.51 to 1.23)	
Antidepressant therapy	No (*n* = 1231)	82.7%	85.8%	53.2%	<0.001	1	<0.001
	Yes (*n* = 257)	17.3%	14.2%	46.8%		5.29 (3.67 to 7.64)	
Benzodiazepine receptor agonists	No (*n* = 1346)	90.5%	91.8%	77.7%	<0.001	1	<0.001
	Yes (*n* = 142)	9.5%	8.2%	22.3%		3.20 (2.05 to 4.99)	
Other psychotropic drugs	No (*n* = 1423)	95.6%	96.2%	89.9%	0.001	1	0.001
	Yes (*n* = 65)	4.4%	3.8%	10.1%		2.85 (1.53 to 5.29)	
Substance consumption	No (*n* = 743)	49.9%	50.3%	46.8%	0.719	1	0.721
	Smoking alone (*n* = 190)	12.8%	12.6%	14.4%		1.23 (0.72 to 2.08)	
	Alcohol alone (*n* = 434)	29.2%	29.2%	28.8%		1.06 (0.70 to 1.60)	
	Smoking + alcohol (*n* = 121)	8.1%	7.9%	10.0%		1.36 (0.74 to 2.52)	
Cardiometabolic comorbidities	0 (*n* = 305)	20.5%	20.0%	25.2%	0.032	1	0.035
	1–2 (*n* = 744)	50.0%	49.5%	54.7%		0.88 (0.57 to 1.34)	
	≥3 (*n* = 439)	29.5%	30.5%	20.1%		0.53 (0.31 to 0.88)	
OSAS severity	Mild (*n* = 772)	51.9%	51.5%	55.4%	0.305	1	0.308
	Moderate (*n* = 347)	23.3%	23.1%	25.2%		1.01 (0.66 to 1.54)	
	Severe (*n* = 369)	24.8%	25.4%	19.4%		0.71 (0.45 to 1.13)	
Insomnia disorder	No (*n* = 572)	38.4%	40.2%	21.6%	<0.001	1	<0.001
	Short sleep duration alone (*n* = 308)	20.7%	21.7%	11.5%		0.99 (0.53 to 1.85)	
	Insomnia without short sleep duration (*n* = 362)	24.3%	23.0%	36.7%		2.96 (1.85 to 4.75)	
	Insomnia with short sleep duration (*n* = 246)	16.6%	15.1%	30.2%		3.72 (2.27 to 6.10)	
Sleep movement disorders	No (*n* = 1178)	79.2%	79.1%	79.9%	0.977	1	0.977
	Moderate to severe PLMs alone (*n* = 90)	6.0%	6.1%	5.8%		0.94 (0.44 to 1.99)	
	RLS alone or combined with PLMs (*n* = 220)	14.8%	14.8%	14.4%		0.96 (0.58 to 1.58)	
EDS	No (*n* = 873)	58.7%	59.7%	48.9%	0.014	1	0.015
	Yes (*n* = 615)	41.3%	40.3%	51.1%		1.55 (1.09 to 2.19)	
BDI without item G	≤10 (*n* = 1274)	85.6%	90.3%	40.3%	<0.001	1	<0.001
	>10 (*n* = 214)	14.4%	9.7%	59.7%		13.78 (9.39 to 20.23)	
SI	No (*n* = 1349)	90.7%					
	Yes (*n* = 139)	9.3%					
	Median				Wilcoxon test		
	(P25–P75)						
Age (years)	51 (43–59)		51 (43–59)	51 (41–57)	0.173		
BMI (kg/m^2^)	29.0 (25.8–33.1)		29.0 (25.9–33.0)	29.0 (25.0–33.5)	0.882		
ESS	9 (6–13)		9 (6–13)	11 (7–14)	0.001		
ISI	13 (8–17)		12 (8–17)	16 (13–20)	<0.001		
BDI	3 (1–7)		3 (1–6)	13 (9–18)	<0.001		
BDI without item G	3 (1–7)		3 (1–6)	12 (8–17)	<0.001		

SI = suicidal ideation, BMI = body mass index, OSAS = obstructive sleep apnea syndrome, PLMs = periodic limb movements during sleep, RLS = restless legs syndrome, EDS = excessive daytime sleepiness, BDI = Beck Depression Inventory, ESS = Epworth Sleepiness Scale, ISI = Insomnia Severity Index.

### 3.3. Multivariate Analyses (Table 4)

After hierarchically introducing the significant confounding factors highlighted during the univariate analyses for adjustment, multivariate logistic regression analyses demonstrated that unlike SSD alone (OR 1.24 [95% CI 0.63–2.43]) and CID without SSD (OR 1.45 [95% CI 0.84–2.50]), only CID with SSD (OR 2.24 [95% CI 1.26–3.96]) was associated with a higher likelihood of SI in apneic individuals (*p*-value = 0.047).

**Table 4:** Multivariate analyses (*n* = 1488).

Variables	Model 1 OR Adjusted (CI 95%)	*p*-Value	Model 2 OR Adjusted (CI 95%)	*p*-Value	Model 3 OR Adjusted (CI 95%)	*p*-Value	Model 4 OR Adjusted (CI 95%)	*p*-Value
Insomnia disorder		<0.001		<0.001		<0.001		0.047
No	1		1		1		1	
Short sleep duration alone	1.09 (0.58 to 2.04)		1.15 (0.61 to 2.18)		1.22 (0.64 to 2.32)		1.24 (0.63 to 2.43)	
Insomnia without short sleep duration	2.68 (1.66 to 4.32)		2.19 (1.33 to 3.61)		2.12 (1.28 to 3.50)		1.45 (0.84 to 2.50)	
Insomnia with short sleep duration	4.04 (2.44 to 6.68)		3.14 (1.86 to 5.31)		3.17 (1.87 to 5.36)		2.24 (1.26 to 3.96)	

Model 1 = Model adjusted for gender and age. Model 2 = Model adjusted for gender, age, antidepressant therapy, benzodiazepine receptor agonists, and other psychotropic drugs. Model 3 = Model adjusted for gender, age, antidepressant therapy, benzodiazepine receptor agonists, other psychotropic drugs, and cardiometabolic comorbidities. Model 4 = Model adjusted for gender, age, antidepressant therapy, benzodiazepine receptor agonists, other psychotropic drugs, cardiometabolic comorbidities, excessive daytime sleepiness, and BDI without item G.

## 4. Discussion

In this study, we demonstrated that SIs were a frequent problem (9.3%) in apneic individuals, which seems to confirm that this specific subpopulation presents a higher suicidal risk than the general population [43,44]. However, this prevalence of SI demonstrated in our study seems to be lower than that of the studies by Choi et al. (2015) (20.5%) and Timkova et al. (2018) (20.1%), which could be explained by the application of different selection criteria for apneic individuals in these two studies [8,16]. Indeed, unlike our study, the studies by Choi et al. (2015) and Timkova et al. (2020) excluded apneic individuals treated with psychotropic drugs [8,16]. However, the application of this exclusion criterion in these two studies may have favored an overestimation of SI in their samples of apneic individuals through a selection bias following recruitment only authorized for patients without psychotropic treatment in cases of comorbid psychiatric disorders. Indeed, in the literature, it has been demonstrated that untreated psychiatric disorders remain one of the main risk factors involved in the occurrence of suicidality both for the general population and for some subpopulations [45,46,47,48]. Furthermore, the prevalence of SI highlighted in our study seems to be equivalent to that of the study by Bishop et al. (2018) (9.7%) [7]. However, similar to our study, apneic individuals with psychotropic treatment were not excluded from this study [7], which may have allowed a better estimate of the prevalence of SI by avoiding recruitment only of patients without psychotropic treatment in cases of comorbid psychiatric disorders characterized by higher suicidal risk. Concerning the other aspects of suicidality, given that our study focused only on SI, it is important to report that some studies have also confirmed the existence of evidence in favor of higher risk of self-harm, suicide attempts, and suicide in apneic individuals [20,21,22]. Thus, despite these potential methodological differences for the recruitment criteria used and the type of suicidal behavior studied, apneic individuals seem to have higher suicidality, which justifies a better identification of the factors involved to allow the development of better management strategies for this suicidal risk in this specific subpopulation.

Consistent with available studies [14,15,49,50,51,52], we confirmed that CID and its subtype with SSD are frequent problems in apneic individuals. Indeed, CID was present in 40.9% of apneic individuals in our sample. In addition, among these apneic individuals with CID, 40.5% presented the insomnia subtype with SSD. Moreover, we have demonstrated that unlike SSD alone and CID without SSD, only CID with SSD was associated with a higher likelihood of SI in apneic individuals. However, in order to better understand this high prevalence of CID with SSD and its potential role in the occurrence of SI in apneic individuals, several pathophysiological hypotheses may be proposed. Firstly, in apneic individuals, the development of dysfunctional sleep behaviors involved in the pathophysiology of insomnia disorder may be favored by the occurrence of psychophysiological conditioning induced by repeated nocturnal awakenings related to obstructive respiratory events [53,54]. However, the presence of these dysfunctional sleep behaviors involved in the occurrence of insomnia disorder may be associated with the development and maintenance of chronic sleep deprivation, leading to a reduction in sleep duration [55,56], which could explain the high prevalence of the insomnia subtype with SSD in apneic individuals from our sample. Secondly, based on the data available, there are many elements in favor of a major impact of insomnia disorder in the development of SI [57,58]. Indeed, in insomniac individuals, the occurrence of SI seems to be favored by the presence of some specific pathophysiological mechanisms affecting psychological functioning (mood dysregulation, cognitive inflexibility, feelings of entrapment, cognitive deficits, and impulsivity), some biological systems (serotonergic system dysfunction and hyperactivation of hypothalamic–pituitary–adrenal axis), and the chronotype (alterations of circadian rhythms) [57,58]. Furthermore, sleep duration related to insomnia disorder seems to play a fundamental role in the severity degree of these specific pathophysiological mechanisms favoring the occurrence of SI since compared to insomniac individuals without SSD, those with SSD present a more severe clinical phenotype characterized by more marked psychological, biological, and circadian alterations [59,60,61]. However, the existence in insomniac individuals of these specific pathophysiological mechanisms worsened in cases of SSD could provide a better understanding of the higher likelihood of SI only associated with CID with SSD highlighted in our sample of apneic individuals. Thus, given these different elements, systematic research and adequate treatment of CID with SSD could be an interesting perspective in apneic individuals in order to enable the establishment of better care pathways for individuals at high risk of SI in this specific subpopulation.

The highlighting of this potential role played by CID with SSD in the occurrence of IS for apneic individuals could open up new therapeutic perspectives for the management of suicidality in this specific subpopulation. Indeed, in the literature, there are elements in favor of a positive impact of both pharmacological and non-pharmacological treatments of insomnia disorder on SI [62]. Concerning pharmacological treatments, some studies seem to indicate that in majorly depressed individuals with CID, the association of hypnotic medication with antidepressant treatment or the use of a sedative antidepressant was associated with a reduction in the severity of depressive symptoms and an improvement in SI [63,64,65,66]. Furthermore, regarding non-pharmacological treatments for insomnia disorder, cognitive–behavioral therapy for insomnia currently remains the recommended first-line therapeutic strategy for the management of insomniac individuals [67,68,69,70]. Indeed, cognitive–behavioral therapy for insomnia has demonstrated major effectiveness in improving insomnia complaints but also seems to allow a reduction in depressive symptoms and SI in insomniac individuals [71,72]. However, regarding the choice of the best first-line therapeutic approach for CID with SSD in apneic individuals, cognitive–behavioral therapy for insomnia should be preferred since most pharmacological medications for insomnia disorder may induce a negative impact on nocturnal respiratory dynamics favoring a worsening of OSAS [73,74,75]. Furthermore, alongside this treatment of CID with SSD, it is necessary to adequately treat OSAS in order to avoid the maintenance of residual pathophysiological mechanisms specific to OSAS inducing the persistence of SI in apneic individuals [20]. Regarding these specific aspects related to OSAS, we have highlighted that contrary to the symptomatic severity of OSAS (poor sleep quality and excessive daytime sleepiness), the OSAS severity based on OAHI (mild, moderate, and severe) was not associated with the occurrence of SI in apneic patients from our sample, which seems to be consistent with the limited data available. Indeed, in the study by Timkova et al. (2018) [8], the OSAS severity based on OAHI was not associated with the occurrence of SI, unlike poor sleep quality and fatigue. These elements could indicate that the symptomatic severity of OSAS rather than the OSAS severity based on the OAHI would play a central role in the development of suicidality in apneic patients, which confirms the need to establish an effective treatment for OSAS in order to allow adequate management of its clinical symptoms. Among the currently available treatments for OSAS, continuous positive airway pressure therapy used with adequate adherence seems to be an interesting option given its potential positive impact on the reduction in depressive symptoms and SI in apneic individuals [76,77]. Finally, in addition to these potential new therapeutic strategies for the prevention of suicidality in apneic individuals, it remains important to identify and treat psychiatric comorbidities in this specific subpopulation since the development of SI and the occurrence of suicidal acting remain mainly associated with the presence of psychiatric disorders [78,79,80,81].

A summary of the potential clinical implications of this study for better management of suicidality in apneic individuals is available in Table 5 [62,76,77,82,83,84,85,86].

### 4.1. Limitations

Since the data used were retrospectively extracted from the polysomnographic recordings database of the Brussels University Hospital without direct checks from apneic individuals selected for this study, the realization of prospective complementary studies is essential in order to confirm the results obtained during the analyses performed. In addition, although the cross-sectional design of our study may potentially limit the establishment of a causality between CID with SSD and the occurrence of SI in apneic individuals, it is important to note that our study is the first to address this specific issue in this particular subpopulation, which opens up new perspectives for carrying out additional longitudinal studies that could confirm the preliminary results obtained in our study. Indeed, following this demonstration of a higher likelihood of SI associated with CID with SSD in apneic individuals using logistic regression analyses, this study must therefore be considered as a first step before future research is to be carried out on this problem. On the other hand, in this study, sleep-disordered breathing other than OSAS was an exclusion criterion, which means that our results may only be applied to individuals with OSAS. Furthermore, since we focused only on SI in this study, our results cannot be generalized to other markers of suicidality (suicide attempt and suicide). Finally, the database of the Sleep Laboratory of the Brussels University Hospital only contains apneic individuals who have agreed to undergo a polysomnographic test, which may have favored the occurrence of recruitment bias for this study.

### 4.2. Future Prospects

The development of artificial intelligence is booming both for research and clinical use in psychiatry and sleep medicine [87,88,89,90]. Indeed, machine or deep learning analysis models are increasingly used to develop diagnostic tools for sleep disorders and prediction models to better identify individuals at high risk of suicide [91,92,93,94]. However, despite recent advances in artificial intelligence, available guidelines do not currently include its use in the clinical routine for both the diagnosis of sleep disorders and the screening of suicidality [95,96,97,98]. Although models based on artificial intelligence seem to present promising results [91,92,93,94], the diagnosis of sleep disorders and screening for suicidality always require assessment and confirmation by a healthcare professional to meet clinical recommendations [95,96,97,98]. In this context, it seems essential to continue research associated with artificial intelligence in order to enable the development of diagnostic tools for sleep disorders and the detection of individuals at high risk of suicide that may be implemented in the clinical routine. Indeed, although artificial intelligence cannot currently replace healthcare professionals, its implementation may be complementary to classic clinical practice in order to improve the quality of care for patients [99]. Finally, since there appear to be artificial intelligence-based tools that may diagnose insomnia disorder and predict its involvement in the occurrence of SI in the general population [100,101,102], it might be interesting to develop tools specific to apneic individuals in order to be able to compare the results obtained with these tools to those of our study. This approach could allow a confirmation of our results with a different method and would confirm the complementarity of conventional clinical approaches and artificial intelligence-based tools.

## 5. Conclusions

In this study, we confirmed that apneic individuals are a specific subpopulation characterized by higher suicidality. Indeed, 9.3% of apneic individuals from our sample presented SI, which is higher than for the general population. Furthermore, we demonstrated that unlike SSD alone and CID without SSD, only CID with SSD was associated with a higher likelihood of SI in apneic individuals. The highlighting of this higher likelihood of SI associated with CID with SSD seems to indicate that it could be interesting to systematically research and adequately treat this comorbid sleep disorder in apneic individuals in order to enable better management of suicidality in this specific subpopulation. Finally, future prospective longitudinal studies must be conducted to confirm the preliminary results of our study and to allow the development of new care pathways more specific to apneic individuals.

## Figures and Tables

**Table 5:** Summary of the potential clinical implications of this study for better management of suicidality in apneic individuals.

	Proposed Measures
High prevalence of suicidal ideation in apneic individuals	Systematic screening of suicidality in apneic individuals by the referring healthcare professional
	Systematic assessment of protective or risk factors for suicidal acting
	Systematic research for warning signs of suicide (verbal, behavioral, and psychological)
	Suicide inquiry to determine the level of urgency of the suicidal crisis
	Referral of individuals with suicidality to mental health professionals for additional assessment
Conventional management of suicidal ideation in apneic individuals	Non-pharmacological measures: safety plan intervention, no-suicide contract, activating psychosocial support and psychotherapy
	Pharmacological treatment adapted to the medical and psychiatric clinical picture of individuals
	Psychiatric hospitalization if necessary
Specific management of suicidal ideation in apneic individuals	Adequate treatment of obstructive sleep apnea syndrome (continuous positive airway pressure therapy used with adequate adherence)
	Adequate treatment of comorbid insomnia disorder with short sleep duration
	First-line: cognitive–behavioral therapy for insomnia
	Second-line: pharmacological treatment (hypnotic or sedative antidepressant) if failure or contraindication of cognitive–behavioral therapy for insomnia

## Data Availability

The datasets used and/or analyzed during the current study are available from the corresponding author on reasonable request.

## Supplementary Materials

The following supporting information can be downloaded at: https://www.mdpi.com/article/10.3390/jcm13195907/s1, Annex S1: The description of the outpatient care pathway for the apneic individuals included in this study before their admission to the Sleep Laboratory; Annex S2: The description of self-questionnaires used; Annex S3: The description of the applied polysomnography montage; Annex S4: The description of the stay conditions at the Sleep Laboratory; Annex S5: The description of polysomnographic scoring criteria; Annex S6: The description of the confounding factors included in the univariate analyses. References [103,104] are cited in the Supplementary Materials.
