# Supplementary material for: The Association between Suicidal Ideation and Subtypes of Comorbid Insomnia Disorder in Apneic Individuals
Source: J Clin Med. 2024 Oct 3;13(19):5907. doi: 10.3390/jcm13195907 (PMC11477949; doi:10.3390/jcm13195907)
Supplement: Supplementary file 1 [file jcm-13-05907-s001.zip › jcm-3151997-supplementary.pdf]

Supplementary Data

Annex 1

Description of the outpatient care pathway for the apneic individuals included in this study before their admission to the Sleep Laboratory

These apneic individuals were referred to the sleep laboratory by physicians specialized in sleep medicine after an outpatient consultation during which a preliminary assessment of their complaints related to sleep, their ongoing psychotropic/somatic treatments and their somatic/psychiatric comorbidities was systematically performed in order to allow a first diagnostic hypothesis. The polysomnographic examinations were performed in these apneic individuals to allow an objective assessment of their sleep complaints and confirm the suspicion of OSAS highlighted during the outpatient assessment.

Annex 2

Description of self-questionnaires used

- The Beck Depression Inventory (reduced to 13 items) was used to investigate the presence of depressive symptoms. The 13 items of this scale may be scored from 0 to 3, which means that the total score may vary from 0 to 39. A final score of 0-4 indicates an absence of depressive symptoms, 5-7 mild depressive symptoms, 8-15 moderate depressive symptoms, and ≥16 severe depressive symptoms.

- The Epworth Sleepiness Scale was used to investigate daytime sleepiness. The 8 items of this scale assessing sleepiness in different daytime situations may be scored from 0 to 3, which means that the total score may vary from 0 to 24. A final score greater than 10 indicates excessive daytime sleepiness.

- The Insomnia Severity Index was used to investigate the severity of insomnia complaints. The 7 items of this index may be scored from 0 to 4, which means that the total score may vary from 0 to 28. A final score of 0-7 indicates an absence of insomnia complaints, 8-14 subclinical insomnia complaints, 15-21 moderate insomnia complaints, and 22-28 severe insomnia complaints.

Annex 3

Description of the applied polysomnography-montage

- Two electro-oculogram channels
- Three electroencephalogram channels
- One submental electromyogram channel
- Electrocardiogram
- Pressure cannula to detect the oro-nasal airflow
- Finger pulse-oximetry
- Microphone to record breathing sounds and snoring
- Plethysmographic inductive belts to measure thoracic and abdominal breathing
- Anterior tibialis electrodes

Annex 4

Description of the stay conditions at the Sleep Laboratory

The patients went to bed between 22:00 - 24:00 and got up between 6:00 - 8:00, following their usual schedule. During bedtime hours, the subjects were recumbent and the lights were turned off. Daytime naps were not permitted.

Annex 5

Description of polysomnographic scoring criteria

Obstructive apneas were scored if the decrease in air flow was ≥90% for at least 10 seconds whereas obstructive hypopneas were scored if the decrease in airflow was ≥30% for at least 10 seconds with a decrease in oxygen saturation of 3% or followed by micro-arousal. The obstructive apnea-hypopnea index corresponds to the total number of obstructive apneas and hypopneas divided by the period of sleep in hours.

Periodic limb movements were scored based on the following strict criteria: 1) duration between 0.5 to 10 seconds, 2) interval between 5 and 90 seconds from leg movement onset and 3) movements had to be part of a series of ≥4 consecutive movements meeting these criteria. Periodic limb movement index corresponds to the total number of periodic limb movements divided by period of sleep in hours.

Annex 6

Description of the confounding factors included in the univariate analyzes

After a review of the literature on risk factors for SI [25,26,103,104], the potential confounding factors included in this study were age (categorized: <65 years, ≥65 years), body mass index (categorized: <25 kg/m², ≥25 & <30 kg/m², ≥30 kg/m²), substance consumption (categorized: no, smoking alone, alcohol alone, smoking + alcohol), cardiometabolic comorbidities (categorized: 0, 1-2, ≥3), OSAS severity (categorized: mild, moderate, severe), sleep movement disorders (categorized: no, moderate to severe PLMs, RLS alone or combined with PLMs), Beck Depression Inventory without item G (categorized: ≤10, >10), and as binary variables: gender, antidepressant therapy, benzodiazepine receptor agonists, other psychotropic drugs and excessive daytime sleepiness.
